# Host–Microbiome Interactions: Tryptophan Metabolism and Aromatic Hydrocarbon Receptors after Traumatic Brain Injury

**DOI:** 10.3390/ijms241310820

**Published:** 2023-06-28

**Authors:** Yanming Sun, Shuai Wang, Bingwei Liu, Wei Hu, Ying Zhu

**Affiliations:** Department of Critical Care Medicine, Affiliated Hangzhou First People’s Hospital, Zhejiang University School of Medicine, Hangzhou 310000, Chinajluwangs@163.com (S.W.);

**Keywords:** traumatic brain injury, microbiota–gut–brain axis, tryptophan metabolism, aromatic hydrocarbon receptors, gut microbes

## Abstract

Traumatic brain injury refers to the damage caused to intracranial tissues by an external force acting on the head, leading to both immediate and prolonged harmful effects. Neuroinflammatory responses play a critical role in exacerbating the primary injury during the acute and chronic phases of TBI. Research has demonstrated that numerous neuroinflammatory responses are mediated through the “microbiota–gut–brain axis,” which signifies the functional connection between the gut microbiota and the brain. The aryl hydrocarbon receptor (AhR) plays a vital role in facilitating communication between the host and microbiota through recognizing specific ligands produced directly or indirectly by the microbiota. Tryptophan (trp), an indispensable amino acid in animals and humans, represents one of the key endogenous ligands for AhR. The metabolites of trp have significant effects on the functioning of the central nervous system (CNS) through activating AHR signalling, thereby establishing bidirectional communication between the gut microbiota and the brain. These interactions are mediated through immune, metabolic, and neural signalling mechanisms. In this review, we emphasize the co-metabolism of tryptophan in the gut microbiota and the signalling pathway mediated by AHR following TBI. Furthermore, we discuss the impact of these mechanisms on the underlying processes involved in traumatic brain injury, while also addressing potential future targets for intervention.

## 1. Introduction

Traumatic Brain Injury (TBI) represents a significant health concern, characterised by injury to the intracranial tissue due to direct or indirect external force impact. This may manifest in a variety of pathologies, from skull fractures to brainstem injuries, intracranial hematomas, or brain contusions. The consequence of TBI often results in persistent disability or fatality, contributing to its classification as a leading cause of such outcomes globally. Remarkably, it is estimated that nearly half of the world’s populace will encounter a TBI event in their lifetime. Data from China illustrate the magnitude of this issue, with an excess of one million reported TBI cases annually. Globally, over 50 million individuals are impacted by TBI each year, causing approximately 10 million deaths and nearly 30 million lifelong disabilities, thereby exerting significant socioeconomic pressure [1].

The mechanisms underlying neuroinflammatory responses in TBI—particularly in its acute and chronic phases—are yet to be fully elucidated, despite significant strides in clinical diagnosis, treatment, and injury-related research over recent decades [2]. Such neuroinflammation can intensify the primary injury and has been linked to acute secondary injury post-TBI, as well as chronic neurodegenerative conditions such as Parkinson’s and Alzheimer’s diseases. Post-TBI, complex pathophysiological responses are initiated by the body, involving processes of neuroprotection and repair. However, the overactivation of these responses may foster neurodegenerative lesions. Acute immune and neuroinflammatory responses encompass alterations in microglia, astrocytes, oligodendrocytes, and endothelial cells, culminating in the downstream release of pro- and anti-inflammatory cytokines and chemokines and the recruitment of peripheral immune cells. Although initially neuroprotective and reparative, the persistence of these biological processes may contribute to neurodegenerative lesions [3].

The study of gut flora’s role in brain function modulation has witnessed a substantial surge over the last decade, with mounting evidence—both clinical and preclinical—advocating its role in neurodevelopment, neuroinflammation, and behavioural regulation. Dysregulation of the “microbiota–gut–brain axis,” representing bidirectional communication between the gut flora and the CNS, has been implicated in the pathophysiology of neurological disorders, such as Alzheimer’s disease, autism spectrum disorders, multiple sclerosis, Parkinson’s disease, stroke, and TBI [4]. Furthermore, the normal gut flora can influence microglia proliferation, maturation, and function, displaying sex differences, and maternal gut flora can also modulate the microglial phenotype during embryonic development [5,6]. Importantly, the tryptophan metabolism pathways—serotonin, kynurenine, and indole—are regulated directly or indirectly by gut flora [7]. Pertinently, it has been noted that in TBI, tryptophan undergoes distinct branches of kynurenine metabolism in astrocytes and microglia, resulting in the production of neuroprotective and neurotoxic metabolites, respectively [8].

AhR is a ligand-regulated transcription factor with an evolutionary history exceeding 600 million years [9]. This transcription factor plays crucial roles in a range of physiological and pathological processes and is strongly linked with gut homeostasis, autoimmunity, and CNS neoplasia [10,11]. In the CNS, AhR is found in neurons, astrocytes, microglia, oligodendrocytes, monocytes/macrophages, and brain endothelial cells [12], where they regulate the expression of target genes involved in cell proliferation, metabolism, and immune responses [13]. Recent evidence suggests that the AhR signalling pathway is significantly upregulated following TBI. Furthermore, AhR activation has been found to downregulate the integrity of the blood–brain barrier (BBB) [14,15,16], a factor thought to contribute to neurodegenerative pathologies such as cognitive impairment [17]. Given its role in regulating TBI-induced neurological damage and chronic neuroinflammation, and as a critical component of the “microbiota–gut–brain” axis, AhR presents novel therapeutic opportunities for TBI. In this review, we explore the emerging role of AhR in TBI and its prospective use as a therapeutic target, thereby providing a potential trajectory for disease prevention and treatment. 

## 2. The Microbiota–Gut–Brain Axis in TBI

**Figure 1:**
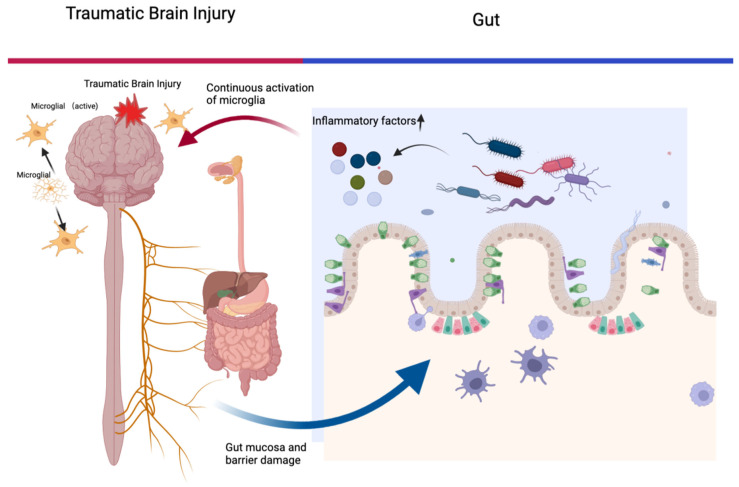
The “microbiome–gut–brain axis” in TBI. primary mechanical injury induces direct microglial activation, while neurotrauma incurs damage to the intestinal mucosa and barrier, provoking alterations in intestinal flora structure and augmenting intestinal permeability. The latter may incite a systemic immune response, where peripheral pro-inflammatory factors stimulate primary injury to the CNS, leading to sustained microglial activation and potentially exacerbating or inciting chronic neuroinflammation.

The Microbiota–gut–brain axis denotes a bidirectional conduit crucial for both CNS and gastrointestinal equilibrium, exerting regulatory influence over a host of functions including gut motility, neurobehavior, visceral pain, and intestinal barrier function [18]^.^ Data from animal models suggest that TBI leads to increased mucosal permeability, which can be observed microscopically within hours after TBI, including oedema, shortening of villi, epithelial shedding and inflammatory inflammation [19]. In the context of TBI, this axis (Figure 1) might operate via several mechanisms: primary mechanical injury induces direct microglial activation, while neurotrauma incurs damage to the intestinal mucosa and barrier, provoking alterations in intestinal flora structure and augmenting intestinal permeability. The latter may incite a systemic immune response, where peripheral pro-inflammatory factors stimulate primary injury to the CNS, leading to sustained microglial activation, and potentially exacerbating or inciting chronic neuroinflammation. However, the precise role of shifts in the structure and functionality of the intestinal flora remains to be fully understood [19].

## 3. Gastrointestinal Dysfunction following TBI

Extant research indicates that TBI patients exhibit a higher propensity towards developing gastrointestinal (GI) disorders compared to healthy controls [20], influencing both morbidity and mortality rates [21,22,23]. One hypothesised mechanism suggests that TBI initiates a stress response that activates the systemic immune system, leading to GI tract dysfunction via autonomic nervous system (ANS) dysregulation [24]. Moreover, TBI effects may be potentiated by psychosomatic stress, exacerbating GI symptomatology [25]. The limited pool of clinical studies examining post-TBI GI symptoms has primarily focused on inpatient complications, specifically food intolerance [26] and GI motility disorders [27], which hamper crucial recovery processes such as nutrition and drug absorption. Approximately half of TBI patients are reported to develop food intolerance, particularly in the week following the injury [26]. Among TBI patients in intensive care units (ICUs), diarrhoea incidences range between 10.5% and 74% [26,28,29]; however, this may not be an exclusive outcome of TBI, considering its high prevalence in critically ill patients undergoing enteral nutrition. Digestive symptoms in TBI patients may be attributed to other factors, including gastrointestinal spasm [27,30] and impaired oesophageal sphincter function [31]. Aligning with clinical study results, murine models of TBI have demonstrated reduced intestinal smooth muscle contractility and delayed GI transit from 1 to 7 days post-injury [32]. The hierarchical neural control of GI motility, particularly in the upper GI tract, involves regulation by the ANS and the enteric nervous system (ENS). Both these systems, adversely affected by TBI, could contribute to early GI motility disorders in hospitalized TBI patients.

## 4. Role of Intestinal Flora in TBI Pathophysiology

**Figure 2:**
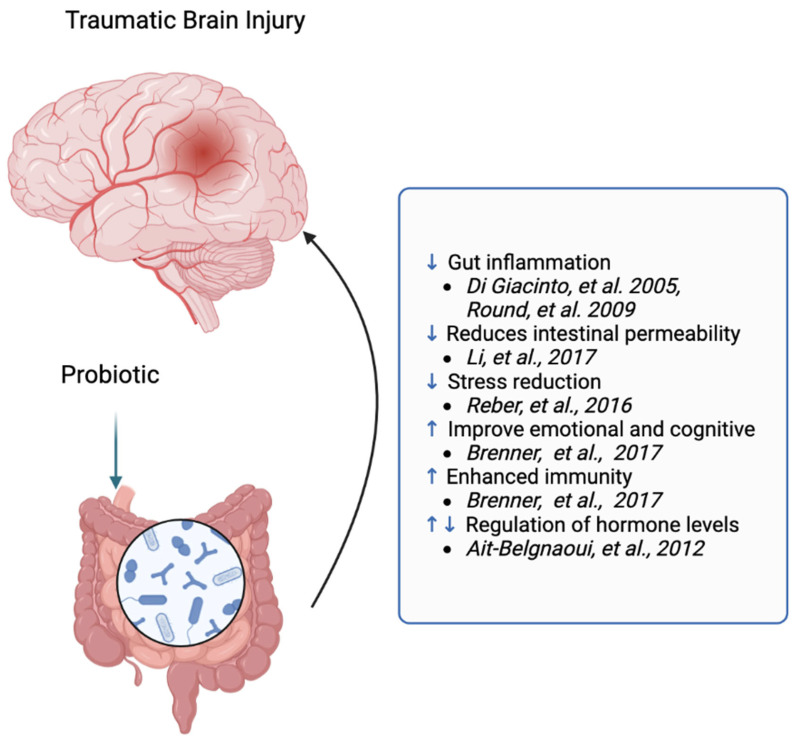
The role of probiotics in traumatic brain injury. ↑: increase;↓: decrease. [38,39,40,41,42,43].

Intestinal flora have been demonstrated to be vital for intestinal immunity, among other aspects (Figure 2). Studies on mice with eradicated intestinal flora highlight the absence of Th17 cells, a variant of IL-17-secreting CD4+ T cells, prominent in the intestinal lamina propria, contributing to the maintenance of intestinal homeostasis through barrier function enhancement, mucin production stimulation, and regulating tight junction function and IgA transport [33]. Germ-free mice displayed reductions in cellular and functional dimensions of the small intestinal immune system, including diminished intra-lymphocytes, lowered IgA levels, smaller Peyer’s patches, decreased CD4+ in the lamina propria and CD8αβ+ cells in the epithelium, and heightened susceptibility to pathogenic bacteria [34,35,36,37].

Recent research identifies dysbiosis as a principal factor exacerbating brain injury through the amplification of intestinal mucosal damage. A potential mechanism is the breach of the intestinal barrier, leading to the transfer of pathogenic bacteria from the gut into the bloodstream. This triggers an aberrant immune response, which exacerbates secondary immune damage to the brain. Hou et al. [44] indicated that the “brain–gut–microbiota” axis might play a pivotal role here. Probiotics can bolster the intestinal barrier, decreasing neurotrauma-induced endotoxemia and quelling systemic immune system dysregulation that could otherwise overstimulate brain microglia. While exhaustive studies concerning probiotic effects on TBI are requisite, probiotics have potential as a therapeutic avenue through increasing IL-10 production and reducing pro-inflammatory cytokines production by intestinal epithelial cells [38,39]. Furthermore, probiotics have been shown to curtail intestinal permeability through modulating the hypothalamic–pituitary–adrenal (HPA) axis. Administration of C. typhimurium to TBI mice demonstrated significant improvements in neurological dysfunction, brain oedema, neurodegeneration, and blood–brain barrier damage, along with notable enhancements in TJ protein expression (Occludin and occluded area 1), p-Akt, and Bcl-2. Probiotic treatment exhibited pronounced effects in reducing plasma lactate and colonic IL-6 levels, upregulating Occludin expression, and preserving the integrity of the intestinal barrier. Moreover, probiotic-treated mice demonstrated increased secretion of intestinal GLP-1 and upregulated expression of GLP-1R in the brain, exerting neuroprotective effects [40]. Hou et al. [44] discovered significantly increased 5-HT concentrations in probiotic-supplemented TBI mice at 1, 3, 7, and 14 days post-operation compared to non-supplemented groups (*p* < 0.05). Brenner et al. [41] reported positive outcomes related to probiotic and prebiotic interventions in a systematic review, including increased regulatory T cells, improved immune regulation, and stress and inflammation reduction. Furthermore, probiotics have been found to mitigate the inflammatory response to post-TBI stress disorder and related symptoms. A recent mouse model study of post-TBI stress disorder [43] revealed a more positive behavioural response in mice supplemented with Mycobacterium vaccae (NCTC 11659), showing its potential to prevent stress-induced colitis and a massive release of anti-CD3-stimulated proinflammatory cytokines, including gamma interferon and interleukin (IL)-6, from freshly isolated mesenteric lymph node cells.

Clinical studies exploring the direct supplementation of probiotics in TBI patients are scarce. Brenner et al. [41] reviewed a handful of clinical studies investigating probiotic and prebiotic interventions in TBI and/or post-traumatic stress disorder patients in a recent systematic review. This review suggested that probiotics or prebiotics improved patient prognosis compared to control groups in three clinical studies involving TBI patients. Current studies have focused on Bifidobacterium supplementation, with animal studies [45,46,47,48] and clinical trials [49] indicating that Bifidobacterium can reduce stress occurrence during the acute phase and promote neurological recovery during the chronic phase. Regarding the duration of administration, Lactobacillus bifidum subspecies, Lactobacillus thermophilus, Lactobacillus bulgaricus, and Lactobacillus subspecies lactis consumption over eight weeks was found to improve affect and cognition. Animal research revealed that two weeks of probiotic consumption correlated with decreased plasma adrenocorticotropic hormone (ACTH) and corticosterone concentrations and diminished hypothalamic adrenocorticotropin-releasing factor (CRF) expression in response to stress [50]. It was also found that probiotics prevented reduced colonic permeability, endotoxemia, and central neuroinflammation [42].

Lastly, targeted enteric antibiotic usage post-TBI can address dysbiosis and produce neuroprotective effects through boosting Treg cell numbers [50]. Broad-spectrum antibiotics can ameliorate neurological prognosis, with Benakis et al. [50] revealing a protective effect of broad-spectrum antibiotics preceding central neurological injury in an ischemic stroke mouse model pretreated with amoxicillin-clavulanic acid for 14 days prior to middle cerebral artery occlusion. This protective effect is transferable through faecal transplantation, linked to neuroinflammation through modulating the pro-inflammatory and inhibitory inflammatory response of intestinal cells from the gut to the nerves. Broad-spectrum antibiotic treatment seems to confer a neuroprotective effect post-TBI. Dennis W. Simon et al. [51] demonstrated reduced associative learning deficits in the fear conditioning test in TBI mice promptly randomized to antibiotics compared to mice without antibiotics (%freeze Cue: 63.7 ± 2.7% vs. 41.0 ± 5.1%, *p* < 0.05) as well as a reduced lesion volume (27.2 ± 0.8 vs. 24.6 ± 0.7 mm^3^, *p* < 0.05). Thus, post-injury antibiotic treatment in a mouse TBI model, and the ensuing depletion of intestinal flora, correlated with neuroprotection, suggesting that antibiotic treatment can be employed for intestinal flora dysbiosis post-TBI, but this necessitates further animal studies and clinical study evidence, as antibiotic usage may introduce other complications.

In conclusion, intestinal flora play a crucial role in TBI pathophysiology, in maintaining intestinal immunity, and in the homeostasis of the brain–gut axis interaction, and the use of probiotics and antibiotics to regulate intestinal flora is critical in enhancing neurological prognosis.

## 5. Changes in the Composition of the Intestinal Flora after TBI

The critical role of intestinal flora in maintaining brain–gut axis homeostasis and their subsequent involvement in traumatic brain injury (TBI) prognosis is an emerging research focus. Notably, Houlden et al. [52] demonstrated the reduction of specific bacterial populations, such as Bacteroidetes, Porphyromonas spp., Stachybotrys spp., and α-proteobacteria, in TBI-afflicted mice, establishing an apparent connection between TBI severity and gut microbiota composition changes [52]. These alterations may stem from a stress response provoked by tissue injury and secondary changes in brain function. Intriguingly, gut microbiota composition shows dynamic modifications during the progression of TBI, with temporal changes in intestinal microbiota composition noted in stool samples collected before and at multiple intervals post-TBI [53]. A study by Nicholson et al. [53] detected alterations in intestinal flora and loss of species diversity as early as two hours post-TBI in rats. This shift was characterized by a decrease in the relative abundance of typically beneficial bacterial groups from the phylum Firmicutes and an uptick in potential pathogens from the phylum Sclerotiniaceae. These changes were particularly pronounced three days post-TBI, with a significant decrease in α-diversity. Mirroring these findings, Treangen et al. [54] also noticed significant shifts in Marvinbryantia and Clostridiales 24 h post-TBI. Taraskina et al. [55] observed a significant increase in Firmicutes (to 68.0%) and a decrease in Bacteroidetes (to 19.8%) seven days post-TBI in animal models. Specific bacterial species, such as Agathobacterspecies, Faecalibacterium, Paraprevotella spp., and Eubacterium coprostanoligenes, demonstrated considerable reduction, whereas species such as Eubacterium sulci, Marvinbryantia formatexigens, and certain RikenellaceaeRC9 gut group species showed substantial increases.

Through our previous animal model investigations, we noted notable alterations in gut microbiota composition at 7 and 28 days post-TBI. In the absence of intervention, some changes persisted up to 28 days, including a decrease in the relative abundance of Akkermansia, a beneficial microorganism. Its supplementation may ameliorate gut microbiota dysbiosis and improve TBI outcomes [56]. Clinical studies also report marked changes in gut microbiota characteristics in TBI patients, with the largest group constituted by Enterobacteriacea [57]. A small cohort study identified significant differences in gut microbiota composition between severe or moderate TBI patients and healthy volunteers, with increases in Enterococ-cus, Parabacteroides, Akkermansia, and Lachnoclostridium and decreases in Bifidobacterium and Faecalibacterium [44]. Interestingly, changes in intestinal flora persisted for up to a year post-TBI [58], with long-term alterations noted even several years after TBI [58]. These shifts demonstrate the persistent effects of TBI on gut microbiota, with distinct dysbiosis characteristics during the acute and chronic phases.

In conclusion, the dysbiosis of intestinal flora and its potential reciprocal relationship with altered neurological function post-TBI require further exploration. TBI induces a decrease in intestinal flora diversity, changes in the abundance of specific flora, a reduction or depletion of beneficial commensal microorganisms, and an overgrowth of opportunistic pathogens. These alterations may significantly impact host metabolism, with potential manifestations in serum metabolite profiles.

## 6. Changes in Tryptophan Metabolites and AHR after TBI

The enduring dysfunction of the BBB post-TBI is well-documented, characterized predominantly by elevated BBB permeability [3]. The health and disease mediation role of intestinal flora, largely through their metabolites, gains added importance against this backdrop [59]. Microglia serve a vital role in both acute and chronic inflammation following TBI, and studies suggest a link between tryptophan metabolites and microglia activity [60]. Tryptophan, an essential amino acid, uniquely binds to albumin, creating a TRP–albumin complex within the bloodstream [61]. This complex functions as a buffering system, maintaining relatively constant free TRP levels. This unbound tryptophan can traverse the BBB into the brain via a sodium-independent amino acid transport system [62]. The gut microbiota can directly metabolize tryptophan into several molecules, including indole and its derivatives [7]. As a substrate for neurotransmitter 5-hydroxytryptamine synthesis, tryptophan holds significant neurological function [63].

**Figure 3:**
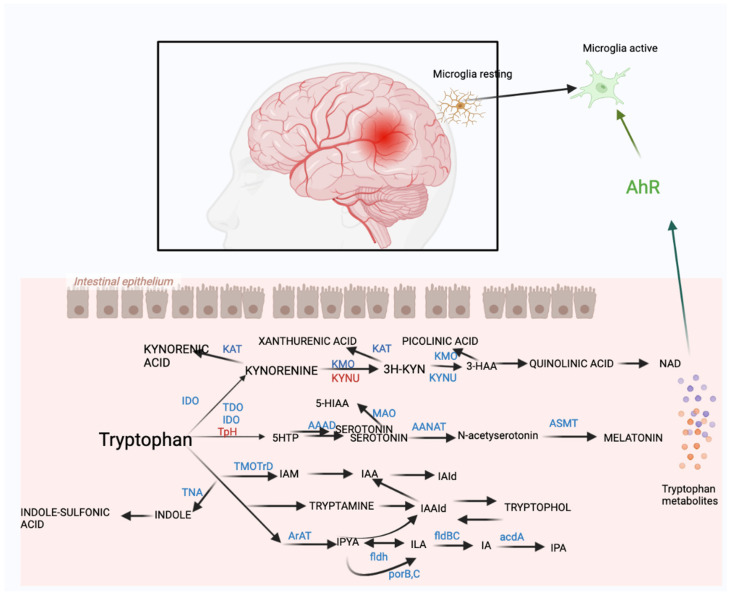
Mechanism of tryptophan metabolism after TBI and on AhR. IDO, Indoleamine 2,3-dioxygenase; TDO, Tryptophan 2,3-dioxygenase; AAAD, Aromatic L-amino acid decarboxylase; MA0, Monoamine oxidases; AANAT, Human arylalkylamine-N-acetyltransferas; ASMT, Recombinant Acetylserotonin-O-Methyltransferase; IPYA, indole-3-pyruvic acid; ILA, indole-3-lactic acid; IA, indole acrylic acid; IAAld, indole-3-acetaldehyde; IEt, indole-3-ethanol (tryptophol); IAM, indole-3-acetamide; KAT, Kynurenine aminotransferase; KMO, Recombinant Kynurenine-3-Monooxygenase; ArAT, Rat aromatic amino acid transaminase; KYNU, Recombinant Kynureninase; TpH, Tryptophan hydroxylase; 5-HT, 5-hydroxytryptamine (serotonin); 5-HIAA, 5-hydroxyindole acetic acid; IAA, indoleacetic acid; KYNA, kynurenic acid; XA, xanthurenic acid.

Post-TBI tryptophan metabolism anomalies tend to manifest within 6–24 h and exhibit temporal variation [64]. Taraskina et al. [55] observed a decrease in serum tryptophan levels three days post-TBI, which normalized by day seven. In a study by Zhang Z. et al. [65], the tryptophan to 5-hydroxytryptamine ratio in rabbit brains showed alterations between days 7 and 21 post-TBI. Similarly, Urban RJ et al. [58] reported significant reductions in tryptophan metabolites in TBI patients even years later. Our team [66] noted altered intestinal metabolism in mice post-TBI and particularly marked changes in tryptophan metabolism at 7 and 28 days post-injury. Specifically, the indole pathway of tryptophan metabolism demonstrated shifts at 7 days post-injury, returning to baseline at 28 days. Thus, TBI results in enduring abnormal tryptophan metabolism, with temporal shifts in various tryptophan metabolic pathways. These changes potentially relate to the acute and chronic trajectory of TBI, though the exact mechanism remains elusive. Therapeutic interventions targeting tryptophan metabolism could influence TBI prognosis and herald novel treatment possibilities (Figure 3).

## 7. Canine Uric Acid Pathway

The kynurenine pathway (KP), which primarily metabolizes the essential amino acid tryptophan in the peripheral and central nervous system, has been implicated in neuroinflammation following TBI through preclinical investigations [8]. Initial catabolism of l-tryptophan to kynurenine (KYN), then to an array of metabolites such as kynurenic acid (KYNA), 3-hydroxykynurenic acid (3-HK), cinnamic acid (CIN), quinolinic acid (QUIN), and picolinic acid (PIC), occurs in intestinal epithelial cells via indoleamine 2,3-dioxygenase 1 (IDO1) and tryptophan 2,3-dioxygenase (TDO) [7,67]. IDO is primarily expressed in macrophages and microglia, while TDO expression is mainly liver-based.

Because the KP serves as the principal endogenous source of NAD+, it plays a vital role in meeting the elevated energy requirements of activated immune cells [68]. Subsequently, KYN is metabolized along one of two distinct branches of KP, producing metabolites with seemingly contrasting characteristics. Kynurenine aminotransferase (KAT), believed to be expressed in astrocytes of the CNS, transforms KYN to kynurenic acid (KynA), a glycine site antagonist of the N-methyl-D-aspartate (NMDA) receptor that competitively inhibits other ionotropic glutamate receptors at high concentrations [69]. These pathways influence microglia activation and play a significant role in chronic neuroinflammation post-TBI.

Post-TBI TRP metabolism through the KP can result in excitatory neurotoxicity due to the release of QUIN and 3HK. Preclinical research has documented a noticeable elevation in kynurenine 3 weeks post-TBI, and a considerable decline in the 5-hydroxytryptamine to tryptophan and melatonin to tryptophan ratios [68]. Zheng et al. [70] observed, in a rabbit TBI model, increased mRNA expression of vital enzymes in the tryptophan-kynurenine pathway, highlighted by significant upregulation of IDO1 mRNA expression in the TBI group at all time points post-injury (*p* < 0.05). Our own investigation [66] unveiled that at 7 days post-TBI, kynurenine significantly rose and KYNA decreased, but by 28 days post-TBI, kynurenine remained unchanged, KYNA increased, and both xanthinic acid (XA) and 8-methoxy-kynurenic acid consistently decreased. The first human evidence of post-TBI abnormalities in KP was from QuinA analysis in the cerebrospinal fluid (CSF) of patients with severe TBI, showing a substantial increase around 72–83 h post-injury [71]. Yan et al. [70] conducted CSF examinations in severe TBI patients, finding that QuinA levels were significantly higher in non-surviving patients when controlled for the time post-injury. An association was found between elevated QuinA concentrations within the first five days post-TBI and a poor six-month prognosis [72]. Similar variations in KP metabolites were seen in TBI patients’ plasma. Chronic severe brain injury patients exhibited extensive KP metabolite disparities, including higher KYN/TRP ratios suggestive of increased IDO activity, and a reduced KynA to KYN ratio [72].

In summary, these findings highlight metabolic irregularities in the KP post-TBI, potentially correlating with disease severity. Interventions targeting pivotal enzymes and metabolites of KP metabolism might constitute a novel therapeutic approach for mitigating chronic inflammation post-TBI.

## 8. Intestinal Inflammation Enhances the Production of Urinary Quinoline and Its Metabolites in Dogs

Zhang et al. [73] intriguingly found that KYN administration in murine primary astrocytes robustly augmented Nod-like receptor protein 2 (NLRP2) expression. This inflammasome activation facilitated the secretion of interleukins IL-1β and IL-18. Moreover, hippocampal administration of KYN in mice elicited astrocytic NLRP2 inflammasome activation and induced depressive-like behaviour. Interestingly, the abrogation of astrocytic NLRP2 inflammasomes mitigated these depressive manifestations. These observations suggest that KYN, capable of breaching the blood–brain barrier, can instigate neuroinflammation, thereby bearing substantial implications for TBI treatment.

Mechanistic insights into the influence of kynurenine metabolism post-TBI are primarily derived from preclinical studies at present. The plasma kynurenine to tryptophan ratio reflects the activity of systemic indoleamine 2,3-dioxygenase (IDO), an enzyme responsive to proinflammatory stimuli and instrumental in fostering immune tolerance [74]. Enhanced IDO activity (kynurenine to tryptophan ratio) and diminished kynurenine transaminase (KAT) activity (KYNA to kynurenine ratio) are indicative of heightened neurotoxic pathway metabolism [75]. Furthermore, tryptophan uptake and metabolism by activated microglia culminate in the generation of neurotoxic metabolites such as 3-hydroxykynurenine (3HK) and quinolinic acid [75].

Tryptophan (TRP), vital for synthesizing all proteins essential for peripheral and central nervous system cell survival, exerts a profound influence on the immune system’s function. Diminished TRP concentrations inhibit peripheral nuclear cell proliferation [76], suppress heterologous immune cell activation [77], and intensify T-cell response suppression [78]. Accordingly, in vivo studies have revealed that blocking the activity of the key TRP-metabolizing enzyme, indoleamine 2,3-dioxygenase-1 (IDO1), in the brain preserves a higher TRP pool, consequently promoting T-cell proliferation [79]. Within the brain, neurons, giant cells, microglia, and infiltrating macrophages metabolize TRP to kynurenine (KYN) via IDO1, which is further metabolized into other neuroactive KP products, including kynurenic acid (KYNA), o-aminobenzoic acid (AA), 3-hydroxykynurenine (3HK), 3-hydroxy-o-aminobenzoic acid (3HAA), and quinolinic acid (QUIN) [80]. This imbalance in the kynurenine pathway represents a potential molecular avenue through which the TBI-induced neurometabolic cascade could precipitate chronic neuroinflammatory responses [8]. Hence, KP metabolites appear to affect TBI-impacted immune cells, such as microglia, thereby influencing the nervous system.

## 9. Indole Pathway

The indole pathway, also known to be perturbed following TBI, exhibits a rise in indole-3-acetaldehyde (IAAld) and indole-3-ethanol (IEt), while a drop in indole-3-lactic acid (ILA) and indole-3-propionic acid (IPA) is observed seven days post-TBI, effectuating a mitigation of neuroinflammation, neurological damage, and cerebral infarction in mice enduring ischemic brain injury [81]. In corroboration, Taraskina et al. [55] discovered a reduction in serum tryptophan commencing 3 days after TBI, which Zhang Z. et al. [68] complemented through documenting a decreased tryptophan to 5-hydroxytryptophan ratio and an interleukin surge in the rabbit brain between 7 to 21 days after-TBI. Although there is a paucity of studies exploring the impacts of indole pathway modulation post-TBI, the pathway’s crucial role in tryptophan metabolism is evident. These alterations in the indole pathway may influence TBI prognosis via the brain–gut axis and tryptophan metabolic pathways.

## 10. 5-Hydroxytryptamine Pathway

Moving to the 5-hydroxytryptamine pathway, intestinal enterochromaffin cells are capable of transforming dietary L-tryptophan into serotonin [82,83]. It is intriguing to note that the gut microbiota appears to govern the synthesis and release of these intestinal enterochromaffin cells [84,85]. For instance, Reigstad et al. [84] demonstrated that short-chain fatty acids (SCFAs) generated by the microbiota fostered serotonin production in human enterochromaffin cells. Tryptophan hydroxylase 1 (TPH1), the rate-limiting enzyme for serotonin synthesis, catalyses the transformation of L-tryptophan into 5-hydroxytryptophan, which is subsequently decarboxylated into 5-hydroxytryptamine (5-HT) or serotonin. Consequently, 5-HT can metamorphose into melatonin, which modulates numerous properties of the gut microbiota, such as oxidative stress and inflammation [86]. Alternatively, 5-HT can be metabolized into 5-hydroxyindoleacetic acid (5-HIAA) [82]. Both 5-HT and 5-HIAA function as AhR signalling agonists. The gut-host microbiota plays a paramount role in the comprehensive biosynthesis of 5-hydroxytryptamine, with more than 90% of human 5-hydroxytryptamine synthesized in the gut [85]. Some bacteria, such as Clostridium ramosum, substantially boost 5-hydroxytryptamine production in intestinal chromogranin cells [87]. It has been established that 5-hydroxytryptophan curtails the severity of intestinal inflammatory responses and immune disorders while modulating gastrointestinal motility and platelet functionality in mice [85]. In the brain, the TPH2 isomer predominantly regulates the synthesis of 5-hydroxytryptamine. It remains unknown if 5-hydroxytryptamine generated in the gut can directly influence 5-hydroxytryptamine transmission in the brain, given the blood–brain barrier’s impermeability to biogenic amines [88]. Fascinatingly, substantial evidence suggests that 5-hydroxytryptamine can modulate immune cell function, thereby controlling inflammation and immunity [89].

## 11. 5-HT Changes after TBI

5-hydroxytryptamine (5-HT) levels rise after TBI, with an observation documented at both seven and twenty-eight days post-injury [66]. Yet, the precise role of 5-HT in TBI remains an enigma. Melatonin, a derivative of 5-hydroxytryptamine, is known to ameliorate intestinal dysbiosis and neuroinflammation [90,91]. Earlier investigations have shown a decrease in melatonin levels seven days post-TBI. Following the breakdown of the blood–brain barrier due to TBI, peripheral 5-HT gains entry into the brain. Ordinarily, the 5-HT selective reuptake transporter (SERT; encoded by the SLC6A4 gene) expunges 5-HT synthesized by enterochromaffin cells from the interstitial space. This crucial molecule, implicated in the regulation of local 5-HT availability, also facilitates the re-uptake of 5-HT in the brain [92]. 5-HT assumes a critical role in the inflammatory response subsequent to secondary brain injury, restraining astrocyte and microglia activation, hence curbing the damage caused by the inflammatory response [92]. A handful of clinical studies suggest that serotonin inhibitors can be effective in treating depression following TBI [93,94,95]. Altered 5-hydroxytryptamine metabolism post-TBI underscores its vital role in the nervous system. It is also suggestive of how its metabolites might influence functions such as chronic inflammation and mood post-TBI, opening up novel therapeutic avenues for TBI treatment.

In summation, the metabolism of tryptophan undergoes modification following TBI, with the metabolic process primarily occurring through three pathways. Notably, one of these involves tryptophan metabolites functioning as ligands for the AHR, a ligand-dependent transcription factor triggered by various synthetic and bioactive molecules. This factor assumes a significant role in immune responses and the mitigation of inflammation [92]. As such, the role of AHR is deemed to be especially consequential.

## 12. Influence of Intestinal Microbiota on Tryptophan Metabolism and AHR following TBI

The AhR performs vital functions within the human organism, with particular significance in barrier organs such as the intestines [96]. AhR plays a pivotal role in maintaining the immune equilibrium in the intestine, thereby contributing to infection defence [96,97]. It also ensures homeostasis of the immune system, especially under conditions of chronic inflammation. Furthermore, AhR signalling promotes neurogenesis. As reported by Wei et al. [98], tryptophan metabolites promote damaged nerve regeneration via AhR, which is an integral part of nerve injury repair after TBI.

Tryptophan metabolites can exert influence on neurological function through AhR. Research indicates that the intestinal microbiota utilizes the messenger role of tryptophan metabolites and SCFA to primarily regulate neural function via AhR [82,99]. Tryptophan metabolites produced by the intestinal flora interact with host cells to activate AhR [100,101]. AhR proteins, which are known to be abundantly expressed in vascular tissue, play a role in neurological disorders [102]. Predominant tryptophan metabolites generated by the microbiota include indole derivatives, for example indole-3-pyruvate, IAAId, leading to the production of KYN metabolites, many of which act as AhR signal activators. This plays a critical role in post-TBI neurological immunity regulation.

The vast majority of dietary tryptophan is absorbed into circulation via the intestinal epithelium, with about one fifth of L-tryptophan being metabolized by the intestinal epithelial cells and microbiota in the intestinal lumen [100]. As previously noted, the intestinal host microbiota can metabolize tryptophan through three main pathways [7]. Certain bacteria within the gut microbiota, such as Lactobacillus spp. and Roussippia spp., contain a tryptophanase enzyme which transforms L-tryptophan into indole-3-pyruvate [103]. This rate-limiting enzyme can be processed into numerous indole derivatives within the gut microbiota as well as in the human liver and kidney. For instance, indole-3-acetaldehyde, indole-3-acetic acid, 3-methylindole, and indole-3-carbaldehyde are important downstream derivatives [103]. Indole-3-pyruvate can also undergo deamination to produce indole-3-propionic acid, a neuroprotective agent [104]. Hendrikx and Schnabl [105] provide a detailed description of the indole pathway of tryptophan metabolism. Notably, indoles serve as important intercellular messengers facilitating communication between bacterial species [106]. Many indole molecules, being potent activators of AhR signalling, can have considerable negative impacts on human health [103]. Hence, it becomes imperative to regulate the gut microbiota following TBI and modulate certain products of tryptophan metabolism. As alluded to earlier, supplementation with probiotics can ameliorate chronic inflammation and enhance TBI prognosis through the modulation of AhR.

## 13. Tryptophan Metabolites as AhR Signalling Activators

AhR was originally perceived as an environmental contaminant sensor. Subsequent studies have found that a large number of gut flora products act as AhR agonists. Specifically, tryptophan metabolites have been identified as potent inducers of AhR signalling [99]. These metabolites play a crucial role in maintaining gut homeostasis and mucosal immunity and, due to their circulation, modulate the brain and other organs. The composition of bacterial strains significantly influences the production of diverse tryptophan metabolites within the intestine. Hubbart et al. [107] delineated the molecular structure and synthetic pathway of indole. Several experiments comparing the effects of different indole derivatives as AhR agonists in cell types found large differences between the different derivatives [99,108,109].

Kynurenine and indole pathways engage in a closely coordinated regulation of gut immunity and homeostasis. For example, AhR promotes the transcription of some tryptophan metabolite genes (IDO1) [110]. Subsequently, the gene activates the KYN pathway, which produces a large number of metabolites. As mentioned earlier, these metabolites can produce neurotoxic or neuroprotective effects. Some metabolites exacerbate the inflammatory response, while others have the opposite property. For example, KYNA enhances AhR sensitivity and thus enhances the anti-inflammatory/immunosuppressive response [111,112]. Fascinatingly, Mezrich et al. [112] illustrated that KYN, but not 3-HK or QUIN, activates AhR signalling in mouse CD4T, DC, and hepatocellular carcinoma cells. They also discovered that KYN/AhR signalling fosters the differentiation of immunosuppressive regulatory T cells (Treg), playing a pivotal role in suppressing inflammatory responses in the intestine and other body regions. Subsequent studies of the metabolites of the KYN pathway revealed that cinnamic acid salt and xanthate are also capable of promoting AhR conduction [100]. This AhR-IDO1-KYN-AhR pathway forms a feedback loop that can be amplified by indole. Correspondingly, other tryptophan metabolites can stimulate AhR signalling [113,114]. Thus, tryptophan substances, after TBI, become potent endogenous AhR agonists, causing acute and chronic lesions after TBI.

## 14. Modulation of Blood–Brain Barrier Post-TBI via AhR

After TBI, BBB integrity is compromised, playing a significant role in acute and chronic neurological deficits following TBI. Interestingly, AhR expression is significantly enriched within BBB cells [115,116]. As previously mentioned, AhR proteins exhibit widespread expression in many barrier organs, suggesting that AhR functions in the brain to regulate the BBB [117]. Several studies have found that AhR activation is associated with BBB disruption. For example, one study [118] showed significant upregulation of AhR within the BBB of TBI mice. AhR signalling amplified the expression of some signals and increased brain oedema. In contrast, in vivo downregulation of AhR factors using CRISPR technology reduced AhR expression, ameliorated BBB disruption, and improved neurobehavioral function. The mechanisms behind AhR-mediated disruption of BBB tight junctions deserve further investigation. Another study [119] demonstrated that excessive AhR activation of indigo sulphate in blood elevated BBB permeability and induced cognitive impairment. The use of indole sulphate in AhR knockout mice did not result in increased BBB permeability or any cognitive impairment, suggesting that the activation of AhR signalling disrupts the integrity of the BBB. It was also shown [120] that it can also lead to a downregulation of β-catenin protein, prompted by its phosphorylation through PKCδ and GSK3β. Another study [119] demonstrated that excessive AhR signalling activation of indigo sulphate in the blood elevated BBB permeability and induced cognitive impairment. They further confirmed these results through showing that indole sulphate in drinking water also led to disruption of the BBB and signs of cognitive impairment. Exposure to indole sulphate in AhR knockout mice did not result in increased BBB permeability or any cognitive impairment, suggesting that the activation of AhR signalling disrupted BBB integrity. It has also been shown [120] that activation of AhR/RhoA signalling induces protein degradation in the brain cells of animals. Hence, reducing phosphorylation could forestall AhR-mediated disruption of cerebrovascular integrity. While some studies are available, the complete mechanisms of AhR signalling and its relationship to BBB disruption post-TBI remain unclear. Nevertheless, the close association between TBI-induced BBB disruption and AhR signalling implies that targeting certain AhR ligands and other pathways may present a potential therapeutic strategy for TBI treatment.

## 15. AhR Signalling Governs Cerebral Blood Flow Post-TBI through the Renin-Angiotensin System

Following TBI, the brain becomes susceptible to oedema, thereby necessitating higher blood pressure to maintain cerebrovascular perfusion pressure. Robust evidence links alterations in AhR signalling to a rise in mean arterial pressure [121,122]. Intriguingly, gut microbiota dysbiosis has been shown to augment arterial blood pressure [123], prompting interest in the potential relationship between gut flora and AhR signalling. Presently, numerous mechanisms are known to govern AhR-driven hoemodynamics, both systemically and within the brain. The renin-angiotensin system (RAS) is important for the regulation of blood pressure. It is recognized that the microbiota can influence the function of this system [124]. All components of the RAS are known to be expressed within the nervous system [125]. Researchers found that [126] the RAS system was found to be AhR-regulated in the neurological microvasculature.

Prorenin converts angiotensinogen to angiotensin I (Ang I), which is subsequently cleaved by the angiotensin-converting enzyme (ACE) to form angiotensin II (Ang II). Several studies [127,128,129] have shown that AhR plays an important role in multiple signalling pathways that mediate blood pressure in mice and is important for maintaining blood pressure homeostasis in the brain. For example, some harmful substances can activate the thrombochondroitin pathway through AhR signalling and damage the BBB barrier [118]. Thus, AhR plays a crucial role in blood pressure regulation following TBI, and certain tryptophan metabolic ligands that modulate AhR contribute to the stabilization of blood pressure, among other functions.

## 16. Perturbed AhR Signalling Engenders Vascular Complications Post-TBI

During the regulation of intracranial blood pressure, nitric oxide (NO) plays a requisite role, with NO production by endothelial NO synthase (eNOS) serving as a crucial vasodilator [129]. eNOS generates NO, which induces the relaxation of smooth muscle cells and fosters vasodilation of cerebral vessels. Evidence suggests that AhR signalling deactivates eNOS enzymes, thereby curtailing NO production and compromising cerebral blood flow (CBF) [130,131]. AhR signalling serves as a potent inducer for reactive oxygen species (ROS) production, for instance, through the stimulation of NADPH oxidase components’ expression [132]. For example, research findings [133] demonstrate that AhR signalling stimulates ROS production and reduces NO. Intriguingly, numerous AhR agonists, such as indole sulphate, have been shown to inhibit NO generation [131,133]. Recently, another study reported [134] a summary of the role of NO in the neurological system. For example, it influences the function of countless other proteins through chemical reactions. Compelling evidence suggests that NO is reduced and contributes to the pathogenesis of atherosclerosis [135,136]. Thus, certain AhR agonists can induce cerebral vascular damage, subsequently impairing vascular endothelial cells, leading to cerebral vasospasm and other causes of cerebral underperfusion. This could induce ischemic injury post-TBI and trigger a secondary injury event.

## 17. Summary

The exploration of gut microbiota in chronic neurological disorders, such as Parkinson’s disease, has emerged as a prominent area of research in recent years. However, studies on tryptophan metabolism in the context of acute and chronic inflammatory responses after TBI are still limited, focusing mainly on preclinical studies. There are no studies on sex and other factors that may contribute to abnormal tryptophan metabolism in TBI [137]^.^ Furthermore, studies probing the inflammatory mechanisms of tryptophan metabolism subsequent to TBI are largely absent. This review delineates the changes in gut microbiota post-TBI, the metabolic dysregulation of tryptophan metabolites, and encapsulates recent studies positing that tryptophan metabolites serve as aryl hydrocarbon receptor (AHR) ligands in glial cells via the disrupted blood–brain barrier, thereby interfering with standard pathways. Moving forward, research will focus on the inflammatory pathways and mechanisms of action of tryptophan metabolites as AHR ligands, acting on glial cells through the compromised blood–brain barrier. This interference with normal pathways leads to the dysregulation of various cytokines and chemokines, thereby amplifying inflammatory responses. Such findings could provide novel treatment options for TBI, ultimately improving its prognosis.
